# Patterns and determinants of the use of complementary and alternative medicine: a cross-sectional study of hypertensive patients in Ghana

**DOI:** 10.1186/1472-6882-14-44

**Published:** 2014-02-04

**Authors:** Irene A Kretchy, Frances Owusu-Daaku, Samuel Danquah

**Affiliations:** 1 Department of Clinical and Social Pharmacy, Faculty of Pharmacy and Pharmaceutical Sciences, Kwame Nkrumah University of Science and Technology, Kumasi, Ghana; 2 Department of Pharmacy Practice and Clinical Pharmacy, College of Health Sciences, University of Ghana School of Pharmacy, Legon, Ghana; 3 Department of Psychology, University of Ghana, Legon, Ghana

**Keywords:** Medication adherence, Traditional medicine, Non-adherence, Side effects, Medication affordability

## Abstract

**Background:**

The use of complementary and alternative medicine (CAM) is widespread and high utilization rates are associated with people who have chronic conditions like hypertension which management requires adherence to conventional treatment. Often however, the use of alternative medicines has been linked to negative health outcomes. The purpose of the study therefore was to evaluate the pattern, determinants and the association between CAM use and the adherence behaviour of hypertensive patients in Ghana.

**Methods:**

A cross-sectional study was conducted using 400 hypertensive patients attending Korle-bu and Komfo Anokye Teaching Hospitals in Ghana from May to July, 2012. Information was gathered on the socio-demographic characteristics of patients, CAM use, and adherence using the 8-item Morisky Medication Adherence Scale (MMAS).

**Results:**

Out of the 400 study participants, 78 (19.5%) reported using CAM with the majority (65.38%) utilizing biological based therapies. About 70% of CAM users had not disclosed their CAM use to their healthcare professionals citing fear and the lack of inquiry by these health professionals as the main reasons for non-disclosure. Males were 2.86 more likely to use CAM than females [odds ratio (OR) = 2.86 (95% CI 1.48 – 5.52), *p* = 0.002]. Participants who could not afford their medications had 3.85 times likelihood of CAM use than those who could afford their medicines [OR = 3.85 (1.15 – 12.5), *p* = 0.029]. In addition, a significant relationship between CAM use and experiences of anti-hypertensive side effects was observed, X^2^ = 25.378, *p* < 0.0001. CAM users were 2.22 times more likely to be non-adherent than participants who did not use CAM [OR = 2.22 (0.70 – 7.14), *p* = 0.176].

**Conclusion:**

Hypertensive patients in Ghana have shown utilization for CAM. It is important that healthcare providers understand the patterns and determinants of CAM use among their patients. Intervention programmes can then be incorporated to enhance the desired health outcomes of patients.

## Background

Complementary and alternative medicine (CAM) encompasses domains of healing resources related to health practices and beliefs different from conventional medicine [1]. Whereas complementary medicine is used in conjunction with standard conventional medical practice, alternative medicine is used as a substitute for conventional medicine [1].

The practice of CAM is gaining ground in both developed and developing countries with utilization pattern as high as 80% recorded in Africa [2]. In Ghana, the word ‘traditional medicine’ (TM) is a more common term for CAM though progressively, these two terminologies are used interchangeably in several other parts of the world [3]. In Ghana, like other developing countries TM is relevant in the health care system and reveals the values, behaviours, and the socio-religious structure of the indigenous Ghanaian society from which it was developed [4]. Although, CAM/TM has been an age long practice in the country, and about 70% of the Ghanaian population depend on it for their health care [5], there is inadequate information on comprehensive policy or guidelines for the preparation, distribution and utilization of this type of practice.

Evidence suggests that CAM use is common among hypertensive patients (8% - 40%) and they use it alongside conventional medical treatments [2,6-8]. Apart from providing cheaper alternatives to conventional medicine, the rise in CAM use has been attributed to its perceived ‘natural’ effect. As a result of this perception, people say it is less harmful and less toxic to the human body [9]. In his review of studies on herbal remedies, non-herbal remedies and other CAM approaches indicated for antihypertensive effect, Ernst (2005) observed generally modest effects of garlic and yoga on hypertension outcomes [10]. Similarly, the rich flavanol content in cocoa has been suggested to be beneficial in relaxing blood vessels and eventually leading to a reduction in blood pressure among people with hypertension. Yet, not all researchers have observed sufficient blood pressure lowering effect in people with essential hypertension [11]. In Ghana, anecdotal evidence from traditional medical practitioners shows the medicinal properties of garlic, natural cocoa powder, moringa and quite recently dandelion in lowering blood pressure. Despite encouraging assertion of CAM use for blood pressure reduction, empirical evidence to draw inferences regarding its effectiveness on blood pressure outcomes remains inadequate [12].

For chronic conditions such as hypertension, health expectations are closely linked with adherence to conventional treatment and the use of alternative medicines may negatively affect health outcomes of patients [13]. Although, there is paucity of information regarding CAM use and adherence to anti-hypertensive medications, poor levels of medication adherence among hypertensive patients has been reported [14]. To improve medication adherence as well as improve health outcomes, it is important to study the trends of CAM use among hypertensive patients in order to inform hypertension management in the country. The purpose of the study therefore was to evaluate the pattern, determinants and effect of CAM use on the adherence behaviour of hypertensive patients.

## Methods

### Study setting

Hypertensive patients were recruited from the medical departments of Korle-Bu Teaching Hospital (KBTH), Accra and Komfo Anokye Teaching Hospital (KATH), Kumasi, in a cross-sectional study. These two Teaching Hospitals were chosen to allow a fair representation of respondents from both southern and northern parts of Ghana, for better generalization of results. The KBTH is the premier and largest teaching hospital in Ghana located in the Accra Metropolitan District of the Greater Accra Region. It is the only tertiary hospital in the southernmost part of Ghana which serves the people of Accra, and surrounding regions in the southern parts of Ghana. The KATH in Kumasi, the Regional Capital of Ashanti Region, is the second-largest hospital in the country and the only tertiary health institution in the Ashanti Region. It is the main referral hospital for the Ashanti, Brong Ahafo, Northern, Upper East and Upper West Regions of Ghana.

### Study participants

A total of four hundred (400) hypertensive outpatients were sampled from the two major teaching hospitals in Ghana. The patients attending KBTH (n = 200) and KATH (n = 200) were recruited using simple random sampling techniques. The minimum sample size was determined using the estimated prevalence of hypertension (28.7%) and a 95% confidence interval [15]. Ghanaian male and female patients diagnosed as hypertensive only or hypertensive with other co-morbid conditions who were eighteen years and above, and reported for treatment at KBTH and KATH were recruited into the study. An additional inclusion criterion was for patients to report prescription of at least one antihypertensive medication. In-patients, pregnant women and incapacitated people were excluded in this study.

### Measures

Data were collected through researcher-administered patient interviews using a standardized questionnaire. The data collection process for 200 hundred participants per study site took place concurrently between May and October, 2012. The questionnaire consisted of sections that assessed patient information such as, age, gender, ethnicity, religious affiliation, marital status, educational level, health insurance, duration of hypertensive diagnosis, the use of CAM, and antihypertensive medication affordability and availability. The Morisky Medication Adherence Scale (MMAS), an 8-item scale, was used to measure medication adherence behavior in hypertensive patients. The scale comprised eight questions about intake of medication, which covered forgetfulness, carelessness and stopping medication taking as a result of either subjectively experiencing an improvement or a deterioration in medical symptoms. Respondents’ scores ranged from < 6, 6 - <8 and 8; these were categorized as low, medium and high adherence respectively. For ease of statistical analysis, patients who scored low and moderate were grouped as poorly adherent [16]. The MMAS was reliable with Cronbach’s alpha of 0.83 in an earlier study of hypertensive outpatients [17].

### Statistical analysis

The Statistical Package for Social Sciences (SPSS) version 20 was used to analyze data for this study. Descriptive statistics were used for frequency counts and percentages of participant characteristics. Chi-square and logistic regression tests were used for evaluating CAM patterns, determinants of CAM use as well as the relationship between CAM use and anti-hypertensive medication adherence.

### Ethical considerations

Ethical clearance was sought from both the Institutional Review Board (IRB) of the Noguchi Memorial Institute for Medical Research and the Committee of Human Research, Publications and Ethics of Komfo Anokye Teaching Hospital. The study protocol and patient consent forms were reviewed, and approved by both committees with ethical approval codes as NMIMR-IRB CPN 044/10-11 and CHRPE/AP/022/12 respectively. The study was strictly voluntary and participants enrolled after informed written consent has been obtained from them.

## Results

### Socio-demographic and clinical characteristics of study participants (Table 1)

**Table 1:** Characteristics of patients

**Variable**	**Frequency**	**Percentage**
**Sex**		
Male	149	37.25
Female	251	62.75
**Age**		
< 20	1	0.25
20 - 29	12	3.00
30 - 39	20	5.00
40 - 49	71	17.75
50 - 59	130	32.50
60 - 69	105	26.25
≥ 70	61	15.25
**Marital status**		
Single	39	9.75
Married	254	63.50
Widowed	73	18.25
Divorced/separated	25	6.25
Co-habiting	9	2.25
**Education**		
No formal	48	12.00
Primary	33	8.25
Secondary	217	54.25
Tertiary	102	25.50
**Religious affiliation**		
Christian (spiritual)	18	4.50
Christian (Charismatic/Pentecostal)	166	41.50
Christian (Orthodox)	174	43.50
Muslim	20	5.00
African traditional religion	4	1.00
Other	18	4.50
**Number of years of having hypertension**		
≤ 10 years	318	79.50
11 – 20 years	49	12.25
21 – 30 years	20	5.00
31 – 40 years	12	3.00
41 – 50 years	1	0.25
**Medication affordability**		
Very affordable	23	5.75
Quite affordable	49	12.25
Somehow affordable	59	14.75
Not so affordable	89	22.25
Unaffordable	180	45.00
**Side effects**		
Low	159	39.75
Moderate	212	53.00
High	29	7.25
**Level of adherence**		
Low	323	80.75
Moderate	50	12.50
High	27	6.75

Low and moderate adherence have been combined as poor adherence.

In terms of patient characteristics, the greatest proportion (32.5%) were 50 – 59 years old, 62.75% were females, 63.5% were married, 54.25% had attained a minimum of secondary school education and 89.5% were Christians. Many of the study participants (82.75%) resided in urban communities and 79.5% had been diagnosed with hypertension for less or equal to 10 years. Although 51.25% of the participants were somehow involved with the purchase of their anti-hypertensive medications, 82% reported various degrees of difficulty with affordability. The experiences of side effects of anti-hypertensive medication were categorized as low (39.75%), moderate (53%) and high (7.25%). In relation to medication adherence, 93.25% poorly adhered to their medications. Without informing their primary health providers, most study participants cut back or stopped taking their medications because they felt worse when they took them, or they felt their blood pressure was under control.

### The pattern of CAM use (Table 2)

**Table 2:** Patterns of the use of CAM

**Variable**	**Frequency**	**Percentage**
**CAM use (N = 400)**		
Yes	78	19.50
No	322	80.50
**CAM type (n = 78)**		
Alternative Medical Practice	16	20.51
Mind & body interventions	10	12.82
Biological Based Therapies (BBT)	51	65.38
Manipulative & body-based methods	1	1.28
Energy therapies	0	0
**Types of BBT (n = 51)**		
‘Bitter leaves’	2	3.92
Moringa	3	5.88
Garlic	5	9.80
Dandylion	4	7.84
Mahogany	3	5.88
Pear leaves	2	3.92
‘Nyame dua’ (God’s tree)	1	1.96
‘Tsotso’	1	1.96
Herbal mixtures	9	17.65
‘Bitter leaves’ + dandylion + moringa	7	13.73
Garlic + dandylion	6	11.76
Dandylion + moringa	5	9.80
Cotton plant	3	5.88
**Source of acquiring BBT (n = 51)**		
Farm/Backyard garden	19	37.25
Family/friends	4	7.84
Purchase from:		
Market	11	21.57
Pharmacy	6	11.76
Herbalist	3	5.88
Gym	3	5.88
General clinic	2	3.92
Herbal clinic	1	1.96
Hawkers	1	1.96
Church	1	1.96
**Disclosure of CAM use (n = 78)**		
Yes	24	30.77
No	54	69.23
Reasons for non-disclosure		
Fear	19	35.00
Lack of inquiry	16	29.60
Not necessary	15	27.80
Just started CAM use	3	5.56
Spiritual matter	1	1.85

Out of the 400 study participants, 78 (19.5%) reported using CAM with the majority (65.38%) utilizing biological based therapies such as dandelion, moringa, garlic, pear leaves, mahogany, ‘bitter leaves’ and herbal preparations (i.e. these are medicinal substances that are essentially extracted from plant parts using unsophisticated methods and presented mostly as liquid or solid dosage forms). None of the participants used energy therapies or bio-electromagnetic based interventions. In acquiring these biological based therapies, 37.25% obtained the products from farms or backyard gardens which may or may not belong to them and 21.57% purchased them from the open market. About 70% of participants had not disclosed their CAM use to their healthcare professionals. These patients did not disclose it because of fear (35%), their health providers had not inquired (29.6%), or they thought it was unnecessary to inform their health providers (27.8%).

### Determinants of CAM use

The determinants of CAM, obtained by using logistic regression analysis, are presented in Table 3. At *p* < 0.05, males had 2.86 times greater odds of using CAM than females. Participants who had difficulty affording their medications had 3.85 times likelihood of CAM use than those who could afford their medicines. In addition, a significant relationship between CAM use and experiences of anti-hypertensive side effects was observed (Table 4). Place of residence, age, marital status, education, employment, number of years of being hypertensive and number of medications did not significantly relate with CAM use. A hierarchical logistic regression model confirmed the significant difference between CAM users and non-users in relation to sex (males) [OR = 2.09 (1.22 – 3.58), *p* = 0.007] and medication un-affordability [OR = 3.7 (1.16 – 11.11, *p* = 0.006].

**Table 3:** Determinants of CAM use (n = 78)

**Variable**	**Odds ratio**	**95% confidence interval**	***p *** **value**
Sex: Male/Female	2.86	1.48 – 5.52	0.002
Residence: urban/rural	0.96	0.45 – 2.04	0.913
Medication unaffordability/affordability	3.85	1.15 – 12.5	0.029
Number of medications taken (1/2+)	0.97	0.73 – 1.29	0.853

**Table 4:** Relationship between CAM use and experiences of anti-hypertensive medication side effects

**Side effects**	**CAM use**	
	**Yes (%)**	**No (%)**
Low	36.7	63.3
Moderate	15.6	84.4
High	55.5	44.8
Chi square test	X^2^ = 25.378, *p* < 0.0001	

### CAM use and effect on anti-hypertensive medication non-adherence

A logistic regression model was used to assess the relationship between CAM use and adherence. CAM users had 2.22 times more likelihood of non-adherence than participants who did not use CAM (Table 5).

**Table 5:** CAM use and effect on anti-hypertensive medication non-adherence

**Variable**	**Odds ratio**	**95% confidence interval**	***p *** **value**
CAM use: Yes/No	2.22	0.70 – 7.14	0.176

## Discussion

With documented evidence suggesting that CAM use is common among hypertensive patients [2,6-8], results from this study revealed that 78 (19.5%) out of the 400 hypertensive patients who participated in the study used CAM alongside conventional medical treatment. This study has shown a relatively lower rate of the use of CAM among study participant than what has been reported in Nigeria [2,18] and UK [7] but higher than a rate of 7.8% in the USA [19].

The perception that biological based products are natural, less toxic, with relatively little or no side effects may have accounted for the significant use among the study participants. The commonly utilized biological based therapies were garlic, moringa, dandelion, ‘bitter leaves’, pear leaves, cotton plant, and herbal preparations obtained from herbalists. Generally, acquisition of such products was quite easy because whereas a majority of participants easily obtained them from farms and backyard gardens, a considerable proportion of patients reported purchasing these readily available products from places such as the market, herbal centres, pharmacies, gym, and the church. For the reason that people get the products easily, it will be difficult for these products to be effectively monitored and controlled by the appropriate regulatory bodies. Moreover, there is the lack of a comprehensive guideline for the preparation, distribution and utilization of CAM practice in Ghana. There is no single, adequately functional body responsible for the control, information, catalogue, and in-depth research of all CAM/TM. Ghana has a Traditional Medicine Practice Act, Act 595 which established a council to regulate the practice of traditional medicine practitioners and license them to practice and to regulate the preparation and sale of herbal medicines [20].Yet, the role of this council is not considerably recognised. Additionally, one of the objectives of the Food and Drugs Authority in Ghana is to regulate all foods, drugs and cosmetics including those that fall under herbal preparations. However, the realization of this mandate is far from optimal and does not include all forms of CAM/TM. A common feature in Ghana is the ease with which people display and sell their CAM/TM products and practices within the community, such as moving from house to house, in public buses and coaches, on the streets, schools and even health centres. Regulating such practitioners and practices becomes quite difficult.

A substantial number of study participants had not disclosed their use of CAM to their healthcare providers, similar to findings from related studies [21,22]. The main reasons for the non-disclosure included fear of health providers getting angry about CAM use and the lack of interest shown by the providers in knowing about the use of alternative remedies among the patients. While noting the relevance of CAM use to hypertensive patients, inadequate information flow between the patient and the healthcare provider concerning the inclusion or substitution of CAM remedies to conventional hypertensive therapies may be problematic in patient management. Such information on CAM use is important for health professionals because they need to assess patient needs in relation to treatment outcomes, medication adherence behaviours, and possible drug interactions. The lack of such monitoring may predispose patients to the risk of developing complications resulting from poor blood pressure control and its associated high incidence of cardiovascular morbidity and mortality.

In this study, the use of CAM in males was significantly higher than in females and males were about three times more likely to use CAM than females. Our findings are consistent with previous studies reporting CAM use dominantly among males [23] and at the same time inconsistent with other studies [2,7,24]. This study also revealed a significant association between the experiences of anti-hypertensive medication side effects and CAM use. Hypertension may contribute to causing sexual dysfunction, and the side effects that characterize many anti-hypertensive medications may worsen erectile dysfunction and other sexual performance difficulties [25]. Sexual activity may differ across cultures and ethnic groups. Issues about sexuality, particularly in Ghana and Africa, constitute cultural constructs that predominantly express masculine worth, dominance and fertility [26,27]. Therefore, it is plausible that the male study participants may have relied on CAM instead of their conventional medicines or used CAM to help alleviate the potential side effects associated with their anti-hypertensive medications. Although the number of people with hypertension is increasing in Ghana, research into sexual function of hypertensive patients is limited. There is therefore the need for further studies on sexual life in relation to anti-hypertensive medications and the subsequent quality of life implications for hypertensive patients.

A very high percentage (93.25%) of the study participants poorly adhered to their medications and this observation is in line with previously reported evidence that medication non-adherence among hypertensive patients in Ghana is 93% [14]. CAM use however did not have a significant relationship with medication non-adherence behaviour. More research is therefore needed in this area to examine possible causes of the extremely high non-adherence behaviour among hypertensive patients. This information would undoubtedly be valuable for intervention programmes to enhance patient medication adherence and health outcomes.

The inability to afford medications was found to be a significant determinant of CAM use. A previous study identified inability to afford anti-hypertensive medications as the main reason for the high medication non-adherence rates among hypertensive patients in Ghana [14]. While the current study does not directly address this trend, the focus was on identifying a potential association between affordability and CAM use which has been noted in past studies as providing cheaper alternatives to hypertension management. The findings from our study suggest that these patients sought less expensive options of hypertension management because they had difficulty acquiring conventional anti-hypertensive medications.

The study acknowledges some limitations. First, although the study participants were recruited from tertiary institutions which serve different health populations, hypertensive patients in Ghana are managed in other health settings. Hence the results may not be generalizable to all hypertensive patients. Second, medication intake information was obtained from the patients who could be prone to social desirability bias.

## Conclusion

The present study has reported CAM use among hypertensive patients in Ghana. The majority of CAM users relied on biological based therapies such as garlic, moringa, dandelion, bitter leaves and herbal preparations. The study observed that males, inability to afford anti-hypertensive medications and side effects of conventional medicines were the main determinants of CAM use. The majority of CAM users had not disclosed their CAM utilization to their healthcare providers. Although no significant association between CAM use and medication adherence was observed, participants who used CAM had a greater likelihood of non-adherence than those who did not use CAM. It is therefore important that healthcare providers understand the patterns and determinants of CAM use among their patients in order to incorporate intervention programmes to enhance health outcomes.

## Competing interests

The authors declare that they have no competing interests.

## Authors’ contributions

IK was involved with research concept, data collection, data analysis, interpretation of results, and writing of manuscript. FO and SD contributed to the research concept and review of manuscript. All authors reviewed and approved the final manuscript.

## Pre-publication history

The pre-publication history for this paper can be accessed here:

http://www.biomedcentral.com/1472-6882/14/44/prepub
